# Identification of a New Transcriptional Co-Regulator of STEAP1 in Ewing’s Sarcoma

**DOI:** 10.3390/cells10061300

**Published:** 2021-05-24

**Authors:** Fatu Badiane Markey, Brigette Romero, Vijay Parashar, Mona Batish

**Affiliations:** 1 Department of Microbiology, Biochemistry and Molecular Genetics, Rutgers Biomedical and Health Sciences, Rutgers University, Newark, NJ 07103, USA; fbadiane@gmail.com; 2 Department of Medical and Molecular Sciences, University of Delaware, Newark, DE 19713, USA; bmromero@udel.edu (B.R.); parashar@udel.edu (V.P.)

**Keywords:** EWSFLI1, STEAP1, single molecule RNA imaging, smFISH, Ewing’s Sarcoma, NKX2.2, co-regulation, ChIP, luciferase assay

## Abstract

Ewing’s sarcoma (ES) is caused by a chromosomal translocation leading to the formation of the fused *EWSFLI1* gene, which codes for an aberrant transcription factor EWSFLI1. The transcriptional targets of EWSFLI1 have been viewed as promising and novel drug targets in the treatment of ES. One such target is six transmembrane epithelial antigen of the prostate 1 (STEAP1), a transmembrane protein that is upregulated by EWSFLI1 in ES. STEAP1 is a hallmark of tumor invasiveness and an indicator of tumor responsiveness to therapy. EWSFLI1 binds to the STEAP1 promoter region, but the mechanism of action by which it upregulates STEAP1 expression in ES is not entirely understood. Upon analysis of the STEAP1 promoter, we predicted two binding sites for NKX2.2, another crucial transcription factor involved in ES pathogenesis. We confirmed the interaction of NKX2.2 with the STEAP1 promoter using chromatin immunoprecipitation (ChIP) analysis. We used single-molecule RNA imaging, biochemical, and genetic studies to identify the novel role of NKX2.2 in regulating STEAP1 expression in ES. Our results show that NKX2.2 is a co-regulator of STEAP1 expression and functions by interacting with the STEAP1 promoter at sites proximal to the reported EWSFLI1 sites. The co-operative interaction of NKX2.2 with EWSFLI1 in regulating STEAP1 holds potential as a new target for therapeutic interventions for ES.

## 1. Introduction

Ewing’s sarcoma (ES) is caused by balanced translocations between the *EWSR1* gene on chromosome 22 and members of the ETS family of transcription factors [1]. The most common translocation partner in ES is the *FLI1* gene on chromosome 11 [2]. The fused *EWSFLI1* gene is translated into a dysfunctional transcription factor, EWSFLI1, that drives the mis-regulation of many oncogenic pathways leading to tumor growth and disease [3]. Targeted therapies to EWSFLI1 are highly desirable but not achievable due to the unstable and internally disordered structure of EWSFLI1 [4]. This disordered fusion protein alters the cells’ transcriptional profile by recruiting to chromatin and binding to promoters and enhancer regions [5,6,7]. It has been shown to interact with GGAA microsatellites, a binding sequence recognized by the FLI1 DNA binding domain in EWSFLI1 [8,9]. However, being unstable, EWSFLI1 engages with chromatin remodeling complexes to gain access to chromatin regions [7,10,11]. Once it gets recruited to the regulatory regions, it relies on other bona fide transcription factors to gain stability and execute its effect [12,13]. Several transcription factors and chromatin remodeling proteins have been identified to assist EWSFLI1 in its function [14,15,16,17,18,19]. These protein partners and the downstream effectors of EWSFLI1 are thus seen as potential therapeutic targets for ES [20,21].

Two of the most critical EWSFLI1 dependent target genes in ES are six transmembrane epithelial antigen of the prostate 1 (STEAP1) and NKX2.2. STEAP1 is a member of the STEAP family of proteins (STEAP1, STEAP2, STEAP3, and STEAP4), all of which are cell-surface proteins with transmembrane domains [22]. The STEAP family of proteins is not expressed highly in most tissues under normal physiological conditions. These proteins have been found to act as oxidoreductases and contribute to oxidative stress, cell growth, inflammation, and differentiation [23]. Of all the members of the STEAP family, STEAP1 is the most well studied. It is a 339 amino acid transmembrane protein implicated in cell adhesion, regulation of reactive oxygen species levels, and acts as an ion channel or transporter for cell–cell communication [24]. STEAP1 expression is highly upregulated in several cancers, including prostate, bladder, pancreas, ovary, gastrointestinal tract, cervix, and testicular cancer, as well as ES [25,26,27,28,29]. In the case of ES, STEAP1 is a direct transcriptional target of EWSFLI1 [25]. Although previous work has shown recruitment of EWSFLI1 to the STEAP1 promoter, it is not known if EWSFLI1 requires assistance from any other transcription factor to execute its effect.

The second critical EWSFLI1 target gene of interest is NK2 homeobox 2 (NKX2.2). NKX2.2 is a member of the developmental sonic hedgehog-GLI1 signaling pathway and is activated in many cancers, including ES [30,31]. NKX2.2 belongs to the NKX family of transcription factors that are expressed during the embryonic development. It is a critical transcriptional regulator and can act as an activator or repressor of genes in the pancreas, CNS, and other organs during development [32]. NKX2.2 and another transcription factor, Neurogenin 3, cooperatively regulate the transcription of Neuro D1, an essential gene in pancreatic islet cells [33]. In ES, NKX2.2 and its upstream proteins, including GLI1 and GLI2, have been studied extensively for their role in tumor progression [16,34,35]. Previous research from Lessnick’s group and others have focused on the requirement of the repressive function of NKX2.2 in promoting ES cancer growth [36]. Previous studies using microarray analyses of NKX2.2 have shown that overlapping sets of genes are dysregulated upon depleting either NKX2.2 or EWSFLI1 in ES [35,37,38]. Specifically, studies have implicated the repressive transcriptional activity of NKX2.2 in maintaining ES oncogenesis [36]. Therefore, NKX2.2 has been hypothesized to play a critical role in the repressive transcriptional effects of EWSFLI1. This theory was further supported by another study utilizing RNA-Seq to demonstrate that EWSFLI1 uses NKX2.2 to repress the mesenchymal features of ES [39]. Therefore, the existing data provide strong evidence that NKX2.2 is a critical player in ES tumor progression [38]. However, the exact mechanism by which NKX2.2 helps coordinate the effects of EWSFLI1 is not well understood.

This study identified a novel partnership between NKX2.2 and EWSFLI1, specifically in coordinating the upregulation of STEAP1 in the ES cells. We found that, in addition to EWSFL1, NKX2.2 also occupies the STEAP1 promoter and is equally important in regulating STEAP1 expression. In addition, NKX2.2 knockdown results in reduced expression of STEAP1 and its downstream factors such as ROS and ROS-dependent genes. Using a luciferase reporter system to examine this partnership in vivo, we confirmed that the presence of both EWSFLI1 and NKX2.2 exert a cooperative effect on STEAP1 expression. Hence, we have identified a novel model for a synergistic and cooperative role of NKX2.2 as a protein partner of EWSFLI1. Elucidating the mechanism of action of NKX2.2 indicates a potential therapeutic target and will provide a better understanding of the biology of ES. Targeting these critical protein-protein interactions is a promising approach for developing specific treatments for Ewing’s sarcoma patients.

## 2. Materials and Methods

Cell lines and cloning: HTB166, TC-71, 6647, A1795, A9423, TC32, and A673 cell lines were cultured in RPMI (Gibco, Waltham, MA, USA, 11875-093) supplemented with 15 % FBS (Millipore Sigma, St. Louis, MO, USA, F2442), glucose (Millipore Sigma, St. Louis, MO, USA, G7021), penicillin/streptomycin (Millipore Sigma, St. Louis, MO, USA, P4333, HEPES buffer (Millipore Sigma, St. Louis, MO, USA, H4034), and L-glutamine (Millipore Sigma, St. Louis, MO, USA, G7513). HEK 293T cells were cultured in DMEM (Millipore Sigma, St. Louis, MO, USA, D6429) supplemented with 10 % FBS, penicillin/streptomycin and L-glutamine SK-ES-1 cell line was cultured in McCoy’s 5A media (ATCC, Manassas, VA, USA, 30-2007) supplemented with 15 % FBS, penicillin/streptomycin and L-glutamine. All cells were cultured at 37 °C with 5% CO2. HTB166 cell lines were purchased from ATCC, and all other ES cell lines were a generous gift from Dr. James Wells, Harvard Medical School. For imaging studies, all cells were grown on 0.1% gelatin (Bio-Rad, Hercules, CA, USA, 170-6537) coated coverslips in 100 cm^2^ Petri dishes until 70% confluent before fixing in 4% PFA (Millipore Sigma, St. Louis, MO, USA, F8775) and permeabilizing in 70% ethanol for hybridization.

A673 and HTB166 knockdown cell lines were designed as previously described [25,36]. Briefly, shRNA targeting NKX2.2 or EWSFLI1 was cloned into the pSuperRetro and pMKO.1 retroviral vector and transfected into 293T EBNA cells for virus production. Empty pSuperRetro vector was used as a negative control. Ewing’s sarcoma cell lines were transduced with virus, and stable cell lines were selected using G418 (Millipore Sigma, St. Louis, MO, USA, A1720) or puromycin (Gibco, Waltham, MA, USA, A11138-03). A673 inducible EWSFLI1 knockdown cell lines were a gift from Dr. Olivier Delattre, Institut Curie [40] and pMKO.1 shNKX2.2 plasmid was a gift from Dr. Stephen Lessnick, Ohio State University [35]. A list of all primers is provided in the Supplementary Table S1.

Single molecule in situ hybridization: Sets of 50 linear oligonucleotide probes, each 20 nucleotides in length, were designed complementary to specific regions of the STEAP1, NKX2.2, FLI1, and EWSR1 mRNA (Biosearch Technologies, Novato, CA, USA). The probes were pooled in equimolar concentrations and conjugated with tetramethylrhodamine (TMR), Alexa Fluor 594 (AF594), or Cy5 fluorophores and purified by high-pressure liquid chromatography, as previously described [41]. Cells were grown on glass coverslips fixed, permeabilized, and hybridized overnight at 37 °C with probes as described in [42]. The following day, coverslips were washed, stained with DAPI, and mounted. A list of probe sequences is provided in Supplementary Table S2.

Fluorescence imaging and analysis: Images were captured on a Zeiss Axiovert 200 M inverted, wide field, fluorescence microscope with a CoolSNAP HQ camera (Photometrics, Tucson, AZ, USA) using OPENLAB software (Perkin-Elmer, Waltham, MA, USA). Z-stack images were captured for each fluorescent wavelength at 2 sec exposure, for a total of 16–20 images, 0.2 mm apart. The compiled z-stack images were analyzed using an in-house designed algorithm with MATLAB software (MathWorks, Natick, MA, USA) to quantify RNA molecules. Each imaging experiment was performed three times, and at least 100 cells were counted for each experiment. The numbers present average molecules with errors indicating 95% confidence interval. The *p*-values were obtained using Student’s *t*-test.

Chromatin immunoprecipitation (ChIP): ChIP was completed using the Millipore EZ-ChIP kit (Millipore Sigma, St. Louis, MO, USA,17-371) according to the manufacturer’s protocol. Briefly, cells were cross-linked in 1% PFA (Millipore Sigma, St. Louis, MO, USA, F8775) and quenched with 10X glycine (Millipore Sigma, St. Louis, MO, USA, 20-282). After washing in PBS, cells were resuspended in SDS lysis buffer containing protease inhibitor cocktail (Millipore Sigma, St. Louis, MO, USA, P8340). Resuspended cells were sonicated to shear DNA using a Bioruptor. A sample of sheared DNA was run on a 1% agarose gel to confirm fragment sizes ranged between 1000 bp and 200 bp, as indicated by the manufacturer. Sonicated cell lysates were pre-cleared and then incubated overnight with end-over-end mixing at 4 °C with antibodies against FLI1 (BD Pharmingen, Franklin Lakes, NJ, USA, 554266) and NKX2.2 (Santa Cruz, Dallas, TX, USA, F-2, sc-514161). Normal mouse IgG was used as a negative control. Protein Agarose G was added to each aliquot of the antibody-containing lysate for 1 h to pull down complexes. After washing, complexes were eluted in the elution buffer, and cross-linking was reversed. DNA was isolated and purified using spin columns (Millipore Sigma, St. Louis, MO, USA, G1N70-KT), and qRT-PCR (BioRad, Hercules, CA, USA, 1708880) was performed using primers listed in Table S1.

Western blot: Confluent cells were lysed for 30 min in a cold RIPA buffer (Millipore Sigma, St. Louis, MO, USA, R0278-50) containing a protease inhibitor cocktail (Millipore Sigma, St. Louis, MO, USA, P8340). Cell lysates were centrifuged to pellet remaining cell debris, and 25 mg of cleared supernatants were boiled in 5XLaemmli buffer to denature proteins. Denatured samples were run on an 8% SDS-PAGE gel in a running buffer for 2 h. An overnight wet transfer assembly was used to transfer the proteins onto a PVDF membrane (Biorad, Hercules, CA, USA, 1620177). The membrane was blocked for 2 h in 5% milk/Tween PBS and treated overnight with primary antibody against FLI1 (BD Pharmingen, Franklin Lakes, NJ, USA, 554266), NKX2.2 (Santa Cruz, Dallas, TX, USA, F-2, sc-514161), STEAP1 (Santa Cruz, Dallas, TX, USA, H-105, sc-25514), or Actin (Santa Cruz, Dallas, TX, USA, I-19, sc1616). Blots were imaged after treatment for 1 h with anti- rabbit secondary antibody (Santa Cruz, Dallas, TX, USA, sc2030), anti-goat (Santa Cruz, Dallas, TX, USA, sc2020), or anti-mouse (Santa Cruz, Dallas, TX, USA, sc-2031,) using a chemiluminescent reagent. All secondary antibodies were conjugated with horseradish peroxidase (HRP).

One-step qRT-PCR: RNA was isolated from cells lysed in Trizol using the phenol-chloroform extraction method according to the manufacturer’s protocol (Millipore Sigma, St. Louis, MO, USA, T9424). RNA concentration and purity were measured on a Nanodrop, and gene expression was analyzed using Qiagen One-Step PCR kit (Qiagen, Hilden, Germany, 210212) according to the manufacturer’s protocol. A list of all primers is provided in the Supplementary Table S1.

DHE, MitoROS, and MitoMass flow cytometry: Reactive oxygen species and mitochondrial cell mass was determined in ES cell lines as previously described [13]. Briefly, cells were cultured on a 12 well plate until 40% confluent and collected after brief treatment with EDTA. Suspended cells were incubated with 50 mM dihydroethidium (Invitrogen, Carlsbad, CA, USA, D1168) according to the manufacturer’s protocol, washed 1X in HBSS buffer (Gibco), and fluorescence was measured by Accuri C6 flow cytometer. Mitochondrial ROS and mitochondrial mass were measured in ES cell lines by incubating suspended cells in 75 mM MitoSOX (Invitrogen, Carlsbad, CA, USA, 1802090) or MitoTracker (Invitrogen, Carlsbad, CA, USA, 1818516) in PBS, according to manufacturer’s protocol. Fluorescence was measured by Accuri C6 flow cytometer.

Luciferase Assay: 293T cells and A673 cells were used for the luciferase reporter assay (Promega, Madison, WI, USA, E665A). Cells were cultured in a 12 well plate until 30–40% confluent. 293T Cells were transfected with plasmids for the expression of EWSFLI1 (pMSCV EWSFLI1), NKX2.2 protein (pCDH NKX2.2), and both at the same time (pMSCV and pCDH) using Lipofectamine 2000 (Invitrogen, 11668019) according to the manufacturer’s protocol. After 24 h, cells were transfected with a luciferase plasmid containing the top region from the STEAP1 transcriptional start site (pGL4.0 -Top) or with the Middle region of STEAP1 promoter (pGL4.0-Middle). Forty-eight hours after initial transfection, cells were trypsinized and harvested. Cell lysate was prepared using the Reporter Lysis Buffer (Promega, Madison, WI, USA, E397A) and lysate was read in a plate reader using the Bright-Glo Luciferase Assay System (Promega, Madison, WI, USA, E2610) according to the manufacturer’s protocol. Lysis buffer and luciferase reagent were used for preparing the negative control.

## 3. Results

### 3.1. NKX2.2 and STEAP1 Expression Are Positively Correlated in Ewing’s Sarcoma Cell Lines

**Figure 1:**
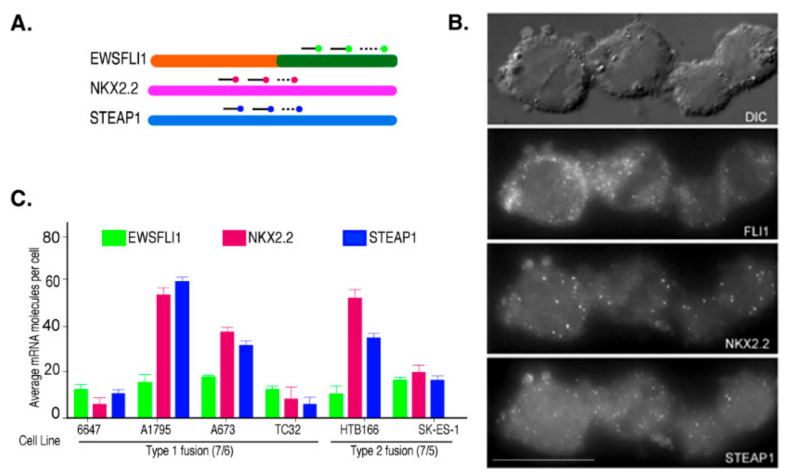
Single-molecule RNA analysis of ES cells: (**A**) A graphical representation of smFISH probes binding to the target RNA. Multiple target RNAs can be imaged simultaneously using different fluorophores for different target RNAs. (**B**) A representative image of HTB 166 cells showing the smFISH signal for EWSFLI1, NKX2.2, and STEAP1 RNAs, where the probes for FLI1, NKX2.2, and STEAP1 mRNAs were coupled with TMR, Texas Red, and Cy5, respectively. The first panel is the bright field image, and the remaining panels are raw images with a merge of 16 z stacks for each filter. (**C**) The average number of FLI1, NKX2.2, and STEAP1 mRNA molecules obtained after counting at least 100 cells for each cell line. The error bars indicate a 95% confidence interval. The scale bar is 5 µm. Each experiment was performed in triplicates.

Ewing’s Sarcoma (ES) is characterized by a chromosomal translocation between the genes *EWSR1* and *FLI1*, which form multiple fusion types based on the breakpoint location. The most common types are type 1 and type 2. Type 1 involves fusion of exon 7 of EWS with exon 6 of FLI1 and is found in 80% of the ES cases, while type 2 fusion includes exon 7 of EWS with exon 5 of FLI1 and is reported in about 15% of ES cases [43]. The type 1 fusion is represented by cell lines 6647, A1795, A673, and TC32, and type 2 fusion is represented by HTB166 and SKES-1. We used single-molecule fluorescence in situ hybridization (smFISH) to explore the heterogeneity in the expression of the fusion transcript EWSFLI1 and its two downstream targets, NKX2.2 and STEAP1, in these cell lines. This method allows single-molecule resolution imaging of these RNAs in individual cells. It helps determine the subtle differences from heterogeneous gene expression that often get lost when analyzing the whole cell populations as in PCR and other high throughput assays [42,44] (Figure 1A). We used probes targeting the 3′ end of FLI1 RNA as a representative of EWSFLI1 since it was previously shown, by other groups and us, that full-length FLI1 RNA is not expressed in the ES cells [11,45]. Interestingly, all cell lines, irrespective of the fusion type, showed similar levels of EWSFLI1 expression (Figure 1B,C). However, most cell lines had either relatively low or relatively high expression of both NKX2.2 and STEAP1. This trend was unique to only NKX2.2 and STEAP1, as EWSFLI1 expression was consistent across all cell lines and did not follow any pattern in relation to NKX2.2 and STEAP1 (Figure 1C).

**Figure 2:**
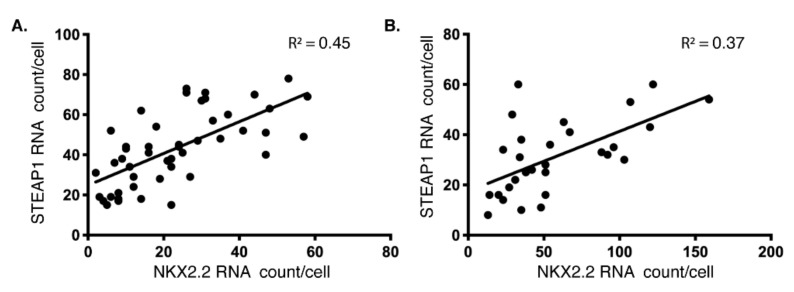
Positive correlation between NKX2.2 and STEAP1 levels: A dot plot with the number of NKX2.2 mRNAs on the X-axis and STEAP1 mRNAs on Y-axis obtained using smFISH in (**A**) type 1 fusion (A1795) and (**B**) type 2 fusion (HTB166) cell lines as described in Figure 1A. Each point on the plot represents a single cell. A linear trendline and R^2^ value are shown on each plot to underscore the positive correlation between these two RNAs’ expressions at a single molecule level in individual cells.

To further confirm the specificity of this correlation, we also imaged single mRNA molecules of EWSR1. We did not see any correlation between the levels of EWSRI with NKX22 and FLI1, indicating the association is specific for NKX2.2 and STEAP1. To analyze this trend further, we plotted the expression of STEAP1 against NKX2.2 mRNA (per cell) from the two ES cell lines, A1795 and HTB166 (Figure 2). As expected, a positive correlation between the expression of these two genes was identified in these cell lines, as cells that had few STEAP1 mRNA molecules also expressed few NKX2.2 mRNA molecules, and cells with many STEAP1 mRNA molecules also had many NKX2.2 mRNA molecules. Since NKX2.2 is a transcription factor, we hypothesized that this association might be due to the role NKX2.2 plays in the regulation of STEAP1.

### 3.2. NKX2.2 and EWSFLI1 Bind within the Same Region of the STEAP1 Promoter

**Figure 3:**
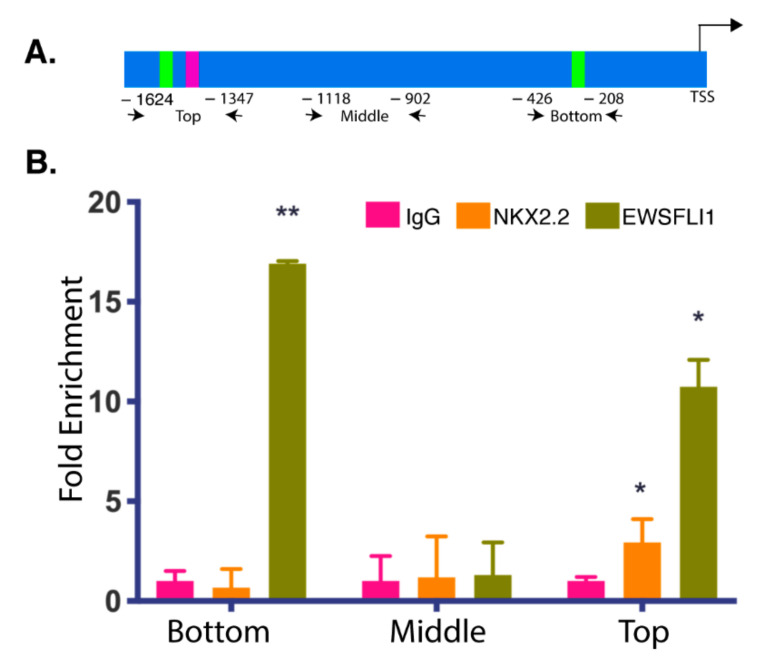
NKX2.2 is associated with the STEAP1 promoter: Chromatin immunoprecipitation (ChIP) of the STEAP1 promoter. (**A**) Graphical representation of binding sites of primers used in ChIP analysis. The numbers represent distance from the transcription start site; not drawn to scale. (**B**) A plot of PCR analyses on the chromatin obtained after pull down with NKX2.2 and EWSFLI1 antibodies from ES cell lysates showing enrichment for both NKX2.2 and EWSFLI1 binding in the top region of the promoter. EWSFLI1 also binds in the bottom region, there is no binding for either transcription factor in middle region. IgG was used as a negative control. Error bars indicate the standard deviation between replicates. * *p* < 0.05, ** *p* < 0.01.

To test the above hypothesis, we scanned the STEAP1 promoter for potential NKX2.2 binding sites. The binding sites for EWSFLI1 on the STEAP1 promoter have already been previously identified using ChIP by Grunwald et al. [25]. The −1465 bp and −250 bp regions from the transcription start site were identified as two binding sites for EWSFLI1 [25]. EWSFLI1 did not bind in the -850 bp region, which was used as a negative control. It has been reported that NKX2.2 recognizes a core sequence of “AAGT” with a consensus sequence (T(t/c)AAGT(a/g)(c/g)TT as reported in the TRANSFAC database [46]. Another core sequence of GAGT has also been reported to be recognized by NKX2.2 [47], with the highest probability value for the octamers -TC AAGT GG and TC GAGT GG. Changes in the flanking regions have been reported to have variable and often non-significant effects on binding prediction [47]. We identified seven occurrences of the core sequence “AAGT” and four occurrences of core sequence “GAGT“ in the STEAP1 promoter (Figure S1). We divided the STEAP1 promoter into three regions: top, middle, and bottom based on the presence of predicted NKX2.2 binding sites and their vicinity to the two known EWSFLI1 binding sites. Upon further analysis of the octamer and consensus sequences, we identified two probable sites for NKX2.2 binding, one in the top and another in the promoter’s middle region. One of these binding sites had the highest probability score and is located right next to the “−1465 bp region” in the top part of the STEAP1 promoter. We performed chromatin immunoprecipitation (ChIP) using antibodies specific for NKX2.2 to confirm if our predicted sites are actual sites for recruitment of NKX2.2 onto the STEAP1 promoter. We also performed ChIP using antibodies specific for EWSFLI1 as a validation control. The pulled-down chromatin was amplified using primers spanning the three selected regions of the STEAP1 promoter, as highlighted in Figure 3A. The top region encompassed the −1465 region, the middle region included sequence around the −850 region and covered the second probable NKX2.2 binding site, and the bottom region covered the −250 bp region reported previously [25]. As expected, EWSFLI1 showed enrichment at the top, and the bottom region, but not in the middle region, consistent with results previously reported [25]. As per our prediction, we found NKX2.2 also showed enrichment in the top region of the STEAP1 promoter; however, we did not see any significant enrichment in the middle and bottom regions (Figure 3B). These results validated our prediction that NKX2.2 is binding directly to the STEAP1 promoter.

### 3.3. NKX2.2 Is Regulating STEAP1 Expression in ES

**Figure 4:**
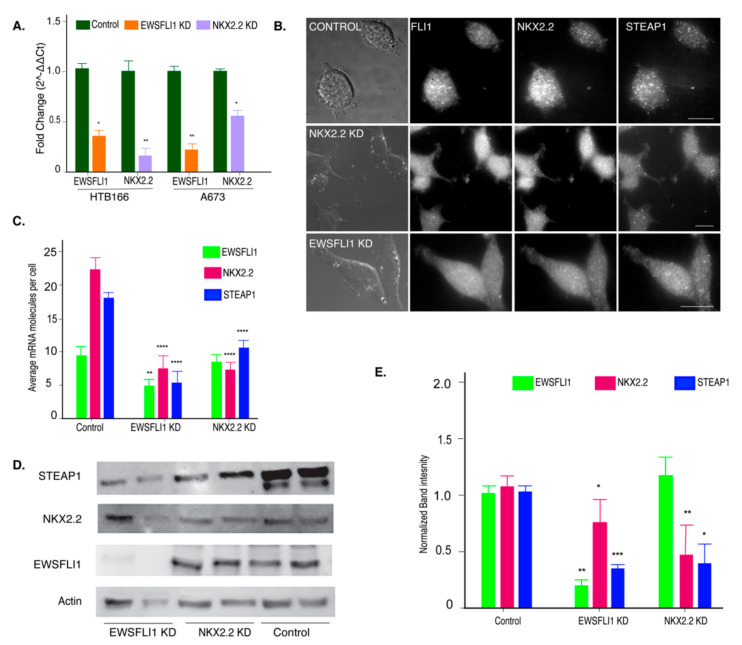
Knockdown of NKX2.2 leads to reduced expression of STEAP1. (**A**) Validation of reduced EWSFLI1 and NKX2.2 gene expression in HTB166 and A673 ES knockdown (KD) cell lines (qRT-PCR). (**B**) The representative image shows the validation of reduced EWSFLI1, NKX2.2, and STEAP1 using smFISH in control and KD cell lines. The probes for FLI1, NKX2.2, and STEAP1 mRNAs were coupled with TMR, Texas Red, and Cy5. The first panel is the bright field, and the remaining panels are raw images with a merge of 16 z stacks for each filter. (**C**) Quantification of smFISH imaging data showing average RNA molecules in individual cells by counting at least 100 cells for each cell line. (**D**) Western blot to determine protein levels of EWSFLI1, NKX2.2, and STEAP1 in control and KD cell lines. Actin was used as a loading control. (**E**) Quantification of the Western blot data. Error bars represent standard deviation. * *p* < 0.05, ** *p* < 0.01, *** *p* < 0.001, **** *p* < 0.0001. The scale bar is 5 µm. The original uncropped Western blot gels are provided in Figure S2.

Previous studies have examined the role of EWSFLI1 on STEAP1 expression using knockdown cell lines, but the role of NKX2.2 in this pathway has not been investigated. We used lentiviral-derived shRNAs to knock down the expression of NKX2.2 in the HTB166 and A673 cell lines. Additionally, the EWSFLI1 knockdown was obtained using doxycycline-based inducible shRNAs against EWSFLI1. Knockdown of NKX2.2 and EWSFLI1 was confirmed by qRT-PCR (Figure 4A). Single-molecule RNA imaging was used to validate the effect of the knockdown on EWSFLI1, NKX2.2, and STEAP1 RNA levels as described in Section 3.1 (Figure 4B,C). The knockdown led to a decrease in the EWSFLI1 and NKX2.2 protein levels in the respective knockdown (KD) cell lines as determined by a western blot analysis (Figure 4D,E). A reduction of STEAP1 expression at both RNA and protein levels was seen in EWSFLI1 and NKX2.2 KD cell lines (Figure 4C,E). These results indicate that both EWSFLI1 and NKX2.2 influence the expression of STEAP1 in ES.

Interestingly, the NKX2.2 knockdown cells express normal levels of EWSFLI1 and still showed a significant reduction in STEAP1 levels (Figure 4E). These results indicated that NKX2.2 could affect the expression of STEAP1 even when EWSFLI1 is present. These results supported our hypothesis that NKX2.2 regulates the expression of STEAP1 for the progression of ES.

### 3.4. NKX2.2 Positively Regulates Reactive Oxygen Species in ES

STEAP1 is a transmembrane protein that modulates the concentration of small molecules, ions, and reactive oxygen species (ROS) within the cell [48]. The elevated level of ROS is a salient feature of aggressive cancers like ES [49,50]. STEAP1 expression has been linked to increased cell proliferation, tumorigenicity, metastasis, and invasiveness in various cancers [22,23,24,27]. Previous studies have shown that STEAP1 overexpression leads to an increase in cytoplasmic and mitochondrial ROS levels in the ES [25].

**Figure 5:**
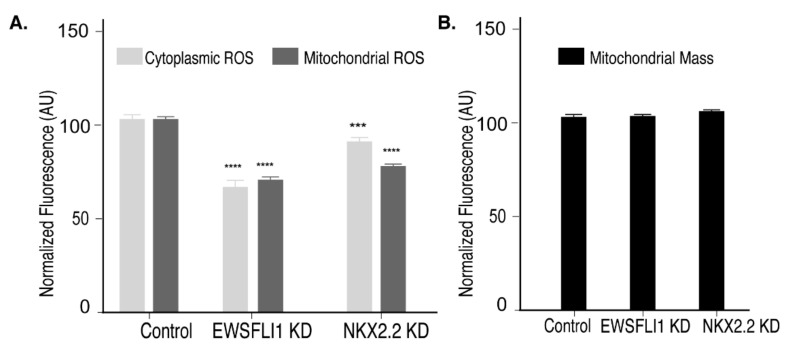
NKX2.2 regulates STEAP1 function: Measurement of cytoplasmic and mitochondrial reactive oxygen species (ROS) in the modified HTB166 cell lines. (**A**) Cytoplasmic ROS was measured using dihydroethidium fluorescence shown as light grey bars. Mitochondrial ROS, shown as dark grey bars, was measured using MitoSox staining. (**B**) The measurement of mitochondrial mass obtained by Mito Tracker green, shown as black bars, to confirm that the mass of mitochondria does not change between control and KD cell lines. The controls (unmodified cell lines) are set as 100%, and the levels in KD cell lines were normalized to that of control. Each experiment was done in triplicate. The error bars represent standard deviation. *** *p* < 0.001, **** *p* < 0.0001.

Since the reduction of NKX2.2 led to a decrease in STEAP1 expression, we wanted to see if this decrease affects the downstream function of STEAP1. Therefore, we tested the levels of cytoplasmic ROS in NKX2.2 KD cells using dihydroethidium fluorescence. We also used EWSFLI1 KD cells as a positive control. We found that both EWSFLI1 and NKX2.2 KD cells showed decreased levels of cytoplasmic ROS. The same trend was observed when we measured mitochondrial ROS levels in these cell lines (Figure 5A). These observed changes were functional as there was no change in the mitochondrial morphology and mass (Figure 5B).

### 3.5. NKX2.2 Regulates the Function of STEAP1

**Figure 6:**
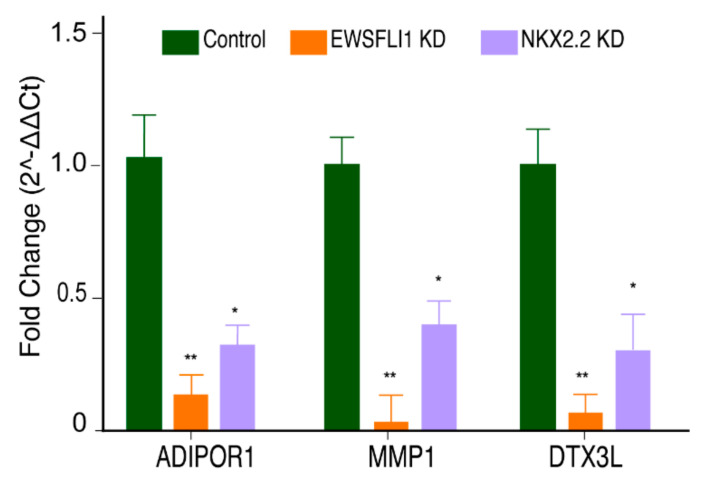
NKX2.2 regulates downstream targets of STEAP1: q-RT-PCR analysis of three downstream targets of STEAP1 in modified HTB166 cell lines. The fold change was calculated by normalizing the expression to a control cell line and with the expression of a GAPDH RNA as a housekeeping reference gene. Each experiment was done in triplicate. The error bars represent standard deviation. * *p* < 0.05, ** *p* < 0.01.

The increase in ROS in ES cell lines from the over-expression of STEAP1 is associated with increased expression of several ROS-dependent genes. The key STEAP1-regulated genes identified to play a role in signaling include Adiponectin receptor 1 (ADIPOR1), Matrix metallopeptidase 1 (MMP-1), and Deltex E3 ubiquitin ligase 3 L (DTX3L) [25]. ADIPOR1 is a receptor for adiponectin, a stimulator of the stem cell proliferation [51,52]. MMP-1 overexpression has been associated with metastasis in numerous cancers [53], and DTX3L has been reported to protect DNA from oxidative stress [54]. A coordinated expression of these three essential genes has been shown to promote the proliferation and invasiveness of ES [25]. Therefore, we looked at the expression of these downstream targets of STEAP1 in the EWSFLI1 and NKX2.2 KD cells. Not only did all three genes show a significantly decreased expression in EWSFLI1 KD cells, a reduction in their expression was also observed in the NKX2.2 KD cells (Figure 6). It is important to note that NKX2.2 KD cells have normal levels of EWSFLI1, further supporting our hypothesis that NKX2.2 is playing a critical role in regulating the expression of STEAP1 and its target genes.

### 3.6. EWSFLI1 and NKX2.2 Act in a Cooperative Manner to Regulate STEAP1

**Figure 7:**
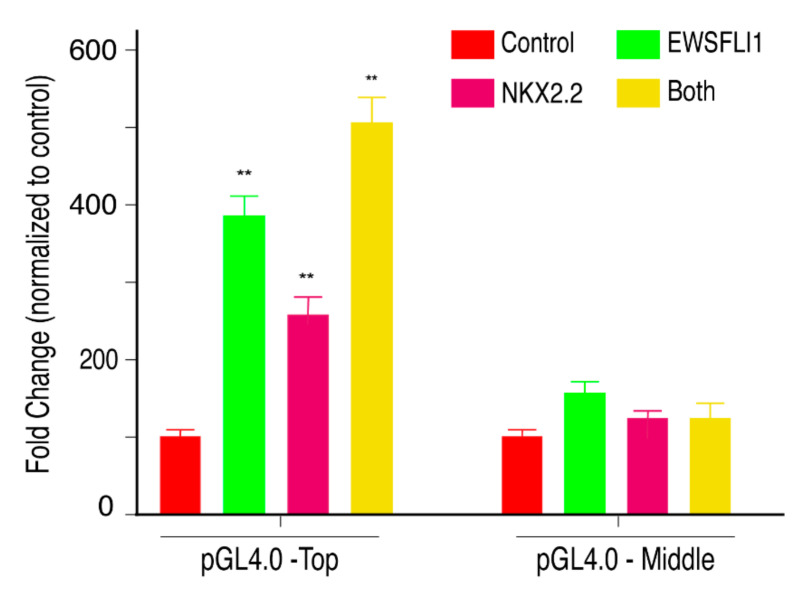
NKX2.2 works co-operatively with EWSFLI1 to regulate STEAP1: HEK293T cells were transfected with an empty vector, or plasmids expressing EWSFLI1, or NKX2.2, or both EWSFLI1 and NKX2.2 expressing plasmids followed by transfection with the pGL4 luciferase plasmid containing STEAP1 promoter regions. pGL4.0-Top contains the top region of STEAP1 promoter that has binding sites for both EWSFLI1 and NKX2.2 while the pGL4.0-Middle contains the middle region of the STEAP1 promoter that does not have any binding site for EWSFLI1 or NKX2.2. Quantification of signal obtained when luciferase substrate was added to the supernatant of lysed cells after normalizing to transfection control. The results were obtained as percent fold change by normalizing the control to 100%. Error bars indicate 95% confidence interval of 3 independent experiments done in triplicates. ** *p* < 0.01.

Since both EWSFLI1 and NKX2.2 showed binding in the STEAP1 promoter at a close regional proximity, and both proteins affect the expression of STEAP1 along with its downstream effector molecules, we wondered if both proteins are acting synergistically to drive STEAP1 expression. To test this in vivo, we cloned the top fragment of the STEAP1 promoter harboring binding sites for EWSFLI1 and NKX2.2 upstream of a luciferase gene in the pGL4.0 luciferase plasmid. We transfected the plasmids expressing EWSFLI1, NKX2.2, or both, in HEK293T cells, which are known to have low endogenous levels of NKX2.2 and no expression of EWSFLI1. The transfection efficiency was confirmed with q-RTPCR using primers specific for NKX2.2 and EWSFLI1 on the cDNA prepared from RNA isolated from these cells (Figure S3). After 24 h of first transfection, STEAP1 luciferase plasmids with either top or middle STEAP1 promoter region were added, eventually followed by a luciferase assay the next day. As expected, in the cells transfected with pGL4.0-Top, we saw an increase in luciferase signal when both EWSFLI1 and NKX2.2 were expressed independently. However, we found that simultaneous expression of both the proteins, significantly increased activity of the luciferase promoter. This increase was not seen when the cells were transfected with pGL4.0-Middle, which is the fragment of STEAP1 promoter that does not have binding sites for EWSFLI1 and NKX2.2. (Figure 7). To further support our hypothesis, we transfected A673 control (wild type) and the A673 NKX2.2 KD cells with pGL4.0- Top and pGL4.0-Middle plasmids. Luciferase assay was carried out 24 h after the transfection. As expected, the A673 NKX2.2 KD cells showed a significant decrease in luciferase expression compared to A673 control cells when pGL4.0-Top construct was expressed. There was no significant difference in the luciferase signal between A673 control and A673 NKX2.2 KD cells when the pGL4.0-Middle construct was expressed (Figure S4). These results confirmed a specific and co-operative effect of the two transcription factors in ES. Thus, supporting our hypothesis that NKX2.2 is acting in synergy with EWSFLI1 to drive STEAP1 expression in ES.

## 4. Discussion

This study’s primary purpose was to fill the missing gaps in understanding disease progression in ES oncogenesis. The inherently unstable nature of EWSFLI1 makes it difficult to develop direct inhibitors for this fusion protein [55]. Therefore, new strategies have been sought to tackle this highly aggressive disease. The research has been focused on either repressing EWSFLI1′s activity by targeting its transcriptional modulators or repressing EWSFLI1 target genes [20]. A handful of drugs have been used in clinical trials for both strategies. For example, YK-4-279 has received an orphan drug status by the FDA; it works by inhibiting EWSFLI1′s interaction with RNA helicase A and prevents its dysregulation of alternative splicing and restores normal helicase activity [56,57,58]. Selective inhibition of direct downstream targets, including Auora Kinase A, GLI1, FOXO1, and mTOR have also shown some promise, particularly when used in combination with other drugs [21,59,60]. NKX2.2 is a direct target of EWSFLI1 and is necessary for oncogenesis but has not been targeted for therapy yet. Our work here shows that NKX2.2 also qualifies as a transcriptional co-regulator of EWSFLI1 in ES. It is the first study where the role of NKX2.2 as a potential partner of EWSFLI1 has been explored. We used smFISH to image RNA distribution in individual cells and found a positive correlation between STEAP1 and NKX2.2 (Figure 2). This was expected since both genes are direct targets of EWSFLI1. However, our ChIP analysis found that NKX2.2 binds directly to the STEAP1 promoter at sites adjacent to the known EWSFLI1 binding sites (Figure 3). Most of the characterization of NKX2.2 in ES and other systems has been focused on its repressive activity, even though NKX2.2 has multiple functional domains, which warrant both repressor and activator roles to this transcription factor [46,61]. In this study, we found an important activator role of NKX2.2 on the STEAP1 promoter. We show that reduced levels of NKX2.2 led to loss of STEAP1 expression even when cells express normal levels of EWSFLI1 (Figure 4). When STEAP1 protein levels are reduced, there is also a clear loss of its key function as an oxidoreductase. This effect from the loss of NKX2.2 is not as pronounced as when EWSFLI1 is reduced (Figure 5). However, it is important to note that EWSFLI1 knockdown also leads to a loss of NKX2.2 expression. Therefore, EWSFLI1 knockdown is considered a double knockdown of both EWSFLI1 and NKX2.2. Additionally, the fact that we saw less dramatic changes in STEAP1 function in terms of ROS levels and the expression of downstream target genes (Figure 5 and Figure 6) implies that NKX2.2 alone is not sufficient to drive the changes in STEAP1 function and expression in ES. Interestingly, STEAP1 was not identified as a target of NKX2.2 in several independent microarrays and RNA-Seq studies [36], indicating that recruitment of NKX2.2 to the STEAP1 promoter is dependent on EWSFLI1. Further, NKX2.2 has been viewed as a downstream effector of EWSFLI1 due to an overlapping set of genes that are regulated when independent data sets for EWSFLI1 and NKX2.2 are compared [39]. It is quite plausible, however, that NKX2.2 is co-regulating these targets instead of acting downstream of EWSFLI1. We have identified STEAP1 as one such co-regulated target by both EWSFLI1 and NKX2.2.

**Figure 8:**
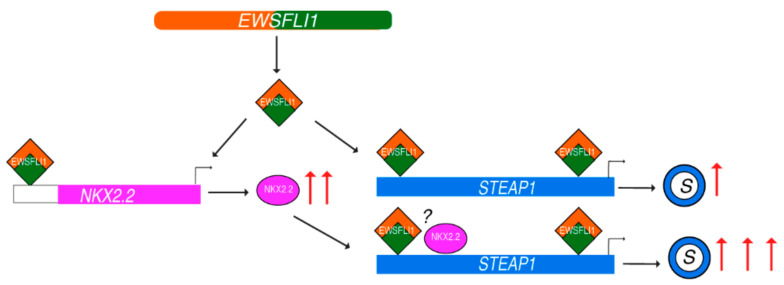
Model representing STEAP1 regulation by NKX2.2 and EWSFLI1: Formation of *EWSFLI1* translocation generates EWSFLI1 fusion protein that binds to STEAP1 promoter and leads to a moderate increase in its expression. EWSFLI1 also leads to upregulation of NKX2.2 by binding to yet unknown enhancer regions. High concentration of NKX2.2 in the cell results in its binding to STEAP1 promoter in proximity to the EWSFLI1. This binding leads to a significant upregulation of STEAP1 (illustrated as a ring with blue outline), which in turn activates reactive oxygen species, and downstream genes contributing to the proliferative and invasive phenotypes of Ewing’s sarcoma. The question mark indicates the unknown nature of interaction between EWSFLI1 and NKX2.2.

Based on our data, we propose a new model for the regulation of STEAP1 in ES. In normal cells, the activity of NKX2.2 at the STEAP1 promoter is low, and with low expression of NKX2.2 in cells, it does not get recruited to the STEAP1 promoter. However, when ES cells express EWSFLI1, it upregulates the expression of a plethora of genes, including NKX2.2. The increased expression of NKX2.2 and possibly an increased accessibility to the STEAP1 promoter region by EWSFLI1 recruitment enables the binding of NKX2.2 to proximal sites on the STEAP1 promoter. The binding of EWSFLI1 and NKX2.2 leads to very high expression of STEAP1 protein and causing a downstream increase in reactive oxygen species and related genes resulting in increased tumor invasiveness (Figure 8).

This model is very plausible as NKX2.2 is a core circuitry complex component and has a proven co-regulatory role in pancreatic cells [33]. Our findings are further supported by a recent report showing that EWSFLI1 regulates and cooperates with core regulatory circuitry (CRC)-dependent transcriptional networks in ES [62]. Shi et al. in their recent work indicated that NKX2.2, upon upregulation, binds to its own promoter, super-enhancers, and the promoters of KLF15 and TCF4 to form an interconnected autoregulatory loop [62]. Our work confirms for the first time that NKX2.2 occupies proximal sites on the STEAP1 promoter, known to be regulated by EWSFLI1. Our data, along with the recently published work, supports the finding that NKX2.2 is acting as a co-regulator of EWSFLI1 transcriptional activity. This work opens several future directions, some of which are ongoing in our lab. First and foremost, it is important to identify what other genes, apart from STEAP1, are co-regulated by EWSFLI1 and NKX2.2. Secondly, it will be interesting to see if these two proteins exist as a complex and have a physical interaction either at promoter regions or independent of chromatin. If the interaction of EWSFLI1 and NKX2.2 is independent of DNA, this could indicate that NKX2.2 can be co-expressed with EWSFLI1 to stabilize and solubilize it, and hence will greatly help in structure–function studies of EWSFLI1. Finally, identification of the co-regulatory role of NKX2.2 makes it a promising drug target of dual value. NKX2.2 is a promising future drug target as well as a reliable biomarker for ES [63]. Developing inhibitors that block NKX2.2 directly can prevent it from co-occupying EWSFLI1 target genes and partnering with EWSFLI1 to stabilize the fusion protein, thus providing a novel avenue for developing targeted ES treatments to tackle this highly aggressive and incurable pediatric cancer.

## Data Availability

Data is contained within the article and Supplementary Materials. The Custom algorithms used for image processing and plasmids generated in the study are available upon request.

## Supplementary Materials

The following are available online at https://www.mdpi.com/article/10.3390/cells10061300/s1, Figure S1: Sequence of STEAP1 promoter, Figure S2: Unmodified Western blot gels, Figure S3: qRT-PCR for transfection efficiency, Figure S4: Luciferase assay in A673 Ewing’s Sarcoma cells, Table S1: list of primers used in the study, Table S2: A list of smFISH probes used in the study.
