# Supplementary material for: Identification of a New Transcriptional Co-Regulator of STEAP1 in Ewing’s Sarcoma
Source: Cells. 2021 May 24;10(6):1300. doi: 10.3390/cells10061300 (PMC8225120; doi:10.3390/cells10061300)
Supplement: Supplementary file 1 [file cells-10-01300-s001.zip › cells-1198070-supplementary.pdf]

Figure S1:

ACTAGAAACAACAGGTGTAAGATTCTATTTCCACCACAAGGTGTCAGTCCACGGTAATT
TAAGCTTCAAA**GGGCTTTAAACTAATCCAAG**GAAAGTCAGTGACTACAGAAAATACTTA
TATTAAAACATGCTTTGTTAAACGGATGCTTGAAGGCGGCATGCTAGTTAAGAGTCATC
ACCACTCCCTAATCTCAAGTACTCAGGGACACAAACACTGCGGAAGGCCGCAGGGTCCT
CTGCCTAGGAAAACCAGAGAACTTTGTTCACTTGTTTATCTGCTGACCTTCCCTCCACT
ATTGTCCTATGACCCTGCCAAATCCCCCTCTG**CGAGAAACACCCAAGAATGA**TCAATAA
AAAAAAATAATAATAATTAAAAAAAAAAAAGTAAAAAAAAAAAAAAAAAAAAAAAAAAA
ACATGAACAGTCTTCGGTGCCCTAGCAGTGAATAAAAAACAATCATGGAAAATGTGGTT
GCGCGTTTATTTTCCTTTGTGTTTTCTACTCAGCTTAGTCCTGAGAATGTTGTATACCA
AATCCCCAGAATTTTGCAATTCATGTCTCAGACCCTATCTTGAGG**CTGGTTCCAACGGA**
GAGAAGTAAATCCCCTGCTCAAGTTGTCTTTTTCTAAAACCTTGGAATATTAACAGTTA
ACTTTACTTTAGGTTTGGAATACTTCATTGAGCTAGGATATGGGAAGCTGCACTTAAAA
GCTAAGGCAAGGAAGGGAGGGACGGAGTAAACATGGGGTTTAAGGGTTTTTTTTTCGGC
TGGGG**AGCAGGGTTTGTGCGATTAA**AGGTACACAATTTGAACAATTCAGGGAGCAAGTC
ACTCTCAGAAATTCAGCTTCGGATTCCTCAAACTGTCAATCACAGCCACTTCAGGACCG
GCTGTTAGGTTTTAAATAAGGTGGCTGGGCGCGGTGGCTCACGCCTGTAATCTCAGCAC
TTTGGGAGGCCGAGGCGGGTGGATCACGAGGTCAGGAATTCAGGACCAGCCTGGCCAAG
ATGGTGAAATCCCCGTCTCTGCTGAAAATACAAAAATTAGCCGGGCACAGTGGCAGGCG
CCTGTAATCCCAGCTACTCGGGAGGCTGAGGCAGGAGAATCGCTTGAACCCGGGGCTGG
AGGTTGCAGTGAGCCAAGATCGCGCCACCGCACTCCAGCCTGGGCAACAGACTGAGACT
CCGTCTCAAAAAAAATAAAAATAAAATAAAAATAAATAAGCTAACGTGTGGGAGGGTTT
GTAAGCTCTAAACGGTCTATCTCAAAGTG**TGATCTGGGAATGCTTCCAT**ATCTCATGGG
AACTTGTTACAAATGAGAATCTCAGACCCCATCCCAGACATACTGAACCGGAAACTGTG
GGCGGACAGCCCAACAATCGGTGTTTTAATAAGCCCCCGGGTAATCTTATACACGCTAA
AGTCTGAGAACCACTGCTCTAGAGAAATGCACAAACGTTCCTTAGTGGAG**ATTAAACAA**
AAGGCGAGGGGTTGGAGGGCGCGAAACGGACCTCAGGTGCAGGGCAGACGGCATTTGAG
AGGAAACTGGGAGAGGCCGGAGTGCGACTTGGGGAGTCTTAGCCTCCAAGGACGTTCCG
GAACCCTGCGGACGCGGGGCGCCAGCAGGTGGCGCTGGACGCGCAACGGACAAGGAGGC
GGGGCCTGCAGCTGGCTTGGAGGCTCCGCGCTCTGGAGGCTC*AGGCGCCGCGTGGGGCC*
CGCACCTCTGGGCAGCAGCGGCAGCCGAGACTCACGGTCAAGCTAAGGCGAAGAGTGGG
TGGCTGAAGCCATACTATTTTATAGAATTAATG

Figure S1: Sequence of STEAP1 promoter: The promoter sequence of STEAP1 showing the sequence of the region before the transcription start site marked with black font and the sequence after TSS is marked in blue italic font with start codon highlighted as yellow. The sequences in red font are the binding sites for the primers for ChIP. The green highlight marks the reported binding sites for EWSFLI1 and the magenta highlight shows the identified probable binding sites for NKX2.2. The boxed magenta region was part of the promoter region that showed enrichment for NKX2.2 in the ChIP analysis. The underlined sequences are the putative core sequences known for NKX2.2 binding. The big black line boxed region at the top marks the region of the promoter used for creating pGL4.0-Top construct and the red line boxed region marks the region of promoter used for creating pGL4.0-Middle construct for the luciferase assay.

Figure S2:

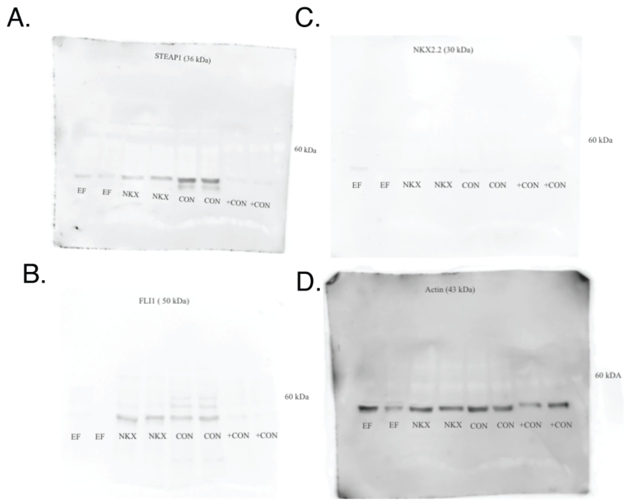

Figure S2: Pictures of uncropped gels for western blot shown in figure 4. The size of each protein is mentioned with its name on top of the gel. (a) The Original western blot for STEAP1 expression; lanes in duplicate = EF KD, NKX KD, knockdown control, positive control for protein, ladder. (B) Original western blot for EWS/FLI1 expression; lanes in duplicate = EF KD, NKX KD, knockdown control, positive control for protein, ladder. (C) Original western blot for NKX2.2 expression; lanes in duplicate = EF KD, NKX KD, knockdown control, positive control for protein, ladder. (D) Original western blot for Actin expression; lanes in duplicate = EF KD, NKX KD, knockdown control, positive control for protein, ladder.

Figure S3:

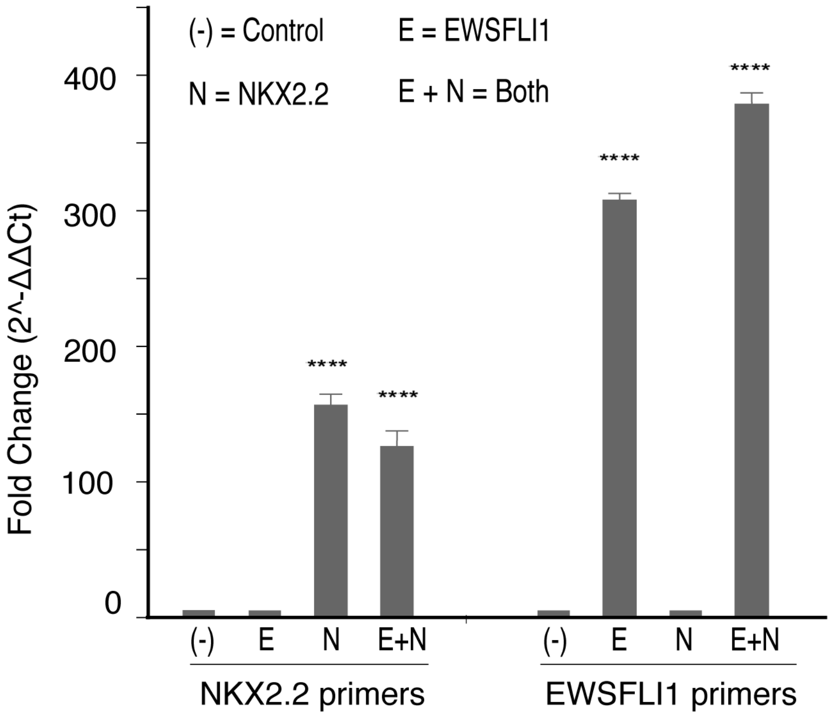

Figure S3: Confirming transfection efficiency for luciferase assay: The HEK293T cells were transfected with plasmids expressing either none or EWSFLI1, or NKX2.2 or both proteins. The cells were lysed 24 hours after transfection and RNA was isolated. 100 ng of RNA from each sample was used to prepare cDNA that was used as template in a qRT-PCR using NKX2.2 and EWSFLI1 and GAPDH primers. The expression was normalized using GAPDH RNA levels. The fold change from the control transfection was calculated using 2^delatdelta Ct. A significant high expression of EWSFLI1 was found in cells transfected with plasmids expressing EWSFLI1 and both proteins and similarly high level of NKX2.2 RNA was found in cells transfected with NKX2.2 and both proteins. Error bars indicate 95% confidence interval from 3 independent experiments **** $p < 0.0001$.

Figure S4

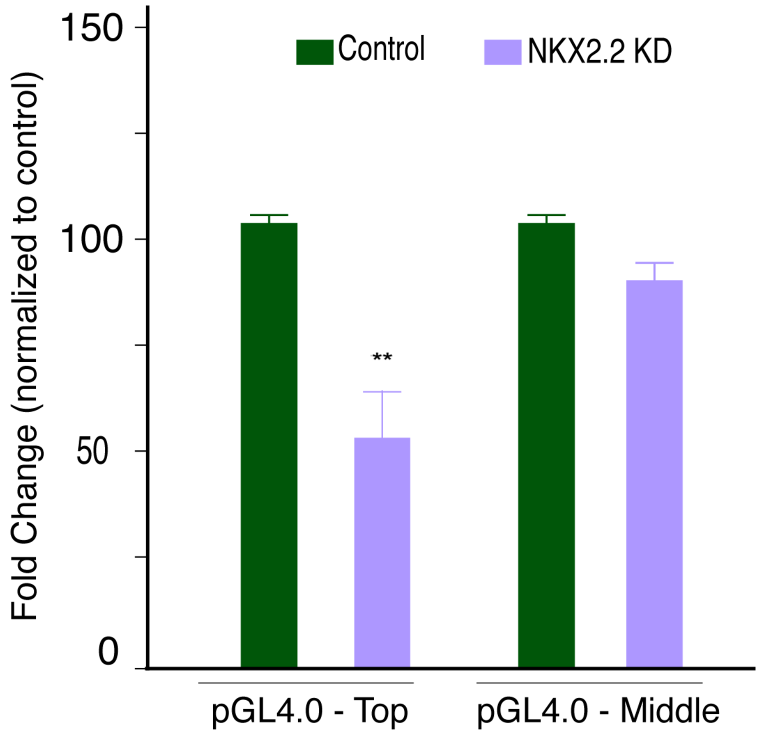

Figure S4: Luciferase reporter assay in A673 Ewing's Sarcoma cells: A673 control (wild type) and A673 NKX2.2 KD cells were transfected with either pGL4.0- Top plasmid (expressing the STEAP1 promoter region with binding sites for both EWSFLI1 and NKX2.2) or pGL4.0- Middle plasmid (STEAP1 promoter region devoid of any binding sites for EWSFLI1 and NKX2.2). Luciferase assay was performed 24 hours after transfection. Quantification of signal obtained when luciferase substrate was added to the supernatant of lysed cells after normalizing to transfection control. The results are depicted as percent fold change by normalizing the signal from the A673 control cells to 100%. Error bars indicate 95% confidence interval of 3 independent experiments done in triplicates. ** $p < 0.01$.

Table S1: List of primers used in the study

Target name	Forward primer	Reverse primer
**STEAP1 Promoter_Top**	GGGCTTTAAACTAATCCAAG	TCATTCTTGGGTGTTTCTCG
**STEAP1 Promoter_Middle**	CTGGTTCCAACGGAGAGAAG	TTAATCGCACAAACCCTGCT
**STEAP1 Promoter_Bottom**	ACCCCATCCCAGACATACTG	GATTACCCGGGGGCTTATTA
**EWS/FLI1**	TAGTTACCCACCCCAAACTGGAT	GGGCCGTTGCTCTGTATTCTTAC
**NKX2.2**	CTCGGTCCTTATGGTGGTTATT	AGTGTTCTCCACTTGCTTTAGA
**STEAP1**	CAATGAGGCGATCCTACAGATAC	CGTCTTCCCAACCATGTCTAAT
**GAPDH**	ACAGTCAGCCGCATCTTCTT	TTGATTTTGGAGGGATCTCG
**ADIPOR1**	GCCATGGAGAAGATGGAAGA	GCTGTGGGGAGCAGTAGAAG
**DTXL3**	GGGCTCTGTGAGTTCTGAGG	TCAGGCAAGTATGCAGTTCG
**MMP-1**	GATGGGAGGCAAGTTGAAAA	CTGGTTGAAAAGCATGAGCA

Table S2: List of probe sequnces used for smFISH imaging

Probe Number	EWSFLI1	NKX2.2	STEAP1
1	cttcgcccgtctaggtcgac	tagtttctaactccaggagg	ttagcttgaccgtgagtctc
2	gtcggagagcagctccagga	cgcggaaatggacgcaggaa	aatagtatggcttcagccac
3	ctcccaggtgatacagctgg	ctctctctgtcttctttgaa	ctgctttccattaattctat
4	gtccgtcattttgaactccc	aaaatgaagcccaacccagt	ttcttggtttgtgatgtctt
5	atgttgggcttgcttttccg	agaaactggggatggggagg	taggcttcattttccaaagt
6	ccggctcagcttgtcgtaat	attggctttaattattggga	tcgtcttcttctaaatttct
7	cactttggtcataatgtttt	cacttggtcaattcgtggcg	tcccgtgtccttatgcaaat
8	tgtaagcatatcttttgccg	ttatgtcgcaaagttgtagc	gtctttttagcatgctggtc
9	ggcaatgccgtggaagtcaa	tcagcgacatggttcgagac	tggtgcaaatgcaaaagcac
10	tcggatgtggctgcagagcc	gaaaaccccgtctttgtgtt	aaattcatcagcatgggctg
11	ggtacttgtacatggacgac	caggtctaagatgtccttga	gctgaagttctgaagggcag
12	acaaagttcaccttctgctg	tcgtagaaggggttcttcag	tgtggaaagagttcctgtgt
13	gaggtccagtattgtgatgc	cgtgcagggagtactgaagg	tattttaattggcaagtgcc
14	cctaagtgtgaaggcacgtg	cttggagcttgagtcctgag	gtcagagatgctataatagc
15	agtaagcttctagtagtagc	ttgtcattgtccggtgactc	cttccctcagaagagtgtaa
16	cttcagctagaaggccactg	ttggagaaaagcactcgccg	gggaagttgctaaagggtga
17	atccagtaagtgtgcaggat	tggaaccagatcttgacctg	tgttgatgaccaggattgga
18	catatgtcctgttgagtcca	cttcatcttgtagcggtggt	agtgatggaaaccattggca
19	ttttgtcttcccttcaaggc	tgagcgcgtgacatggtttg	gcaggtaaaccaatgccaag
20	aaaggttctatccaacaaga	cgctgtaggcagaaaaggga	acaattgctgctatcacacc
21	ctggcccatggaattattca	ttgtactgcatgtgctgcag	acttggttccattatgaagt
22	actgagaattcaaactggca	tagtggagccgagagtcaac	ccaaccaatgtggaaacttc
23	tgcaactcacaagatgctag	tccaaggagacgcaggcaac	tttcttgttaacatccactt
24	tgacaagaaactgctttggt	agagctgggtgggtggaatc	gaaactgagaagcccaaact
25	ttctgtgtcatactgaaccc	ccaaggttcagaaggagagg	attgcatgcagtacagcaaa
26	gaacatgacgggttaagtcc	ctgtaaacacggcgtagagt	cattgggtaagacagactat
27	actggccttacaatcaatgt	agaagcgaagctgcgcaaac	acttgtatctgtaggatcgc
28	attccagttgggtgaaaact	gacgacattaacgctgggac	tgttgatatgcccagtttag
29	ttggcgtcttaaacacacaa	ggtctttttctcgttttcaa	ggcatcttctttattttgtt
30	tgcatactgtccactgagag	aatgcacgaaagcaggggtg	tctccaaacatcatgctcaa
31	tctagagagagtggtcagct	tcgctactcacacaaacata	ccagagacacataaatctcc
32	cccgtctttcaggaatgtat	ctttagaagacggctgacaa	agtattgccaatcccacaat
33	gagtttagaaatccctacaa	tttttaaagtgttctccact	agatgtcacagccaacagag
34	cacagcacttgcgaatctgc	cagaacgtttacatggccat	aagagtcactcacagatgga
35	tggtctgatggtctgacaag	aaagcgaaatctgccaccag	tagtgaaattctctccatgt
36	ttgccttctgattggttggc	tcaccaccgatatttacaac	aattcctagcttgctctgaa
37	ttgtgaccgaggtcaactcg	cctgaaggtcattttggcaa	ttgtgcccagtagaagggaa
38	tgattcgatagtaaaactgc	agaaaggagttggacccaga	ccaggcaaaaatcaatgcgt
39	catacagacagcccaaatgt	cagggttttcttttccatat	tgttttatatctatccactt
40	gtcagtgacttaaacagccc	aatagctgagctccaagttc	gttggaggtgtataccatac
41	gcctgttaaataactgtacc		catggcaggaatagtatgct
42	tatcttttccctgcttgtgt		atcttcagtatcttcttcct
43	tgtttttcccagagctgtga		taattttggtgacgtcttcc
44	tctccacaaggaaagtttc		actgggaacatatctcagtt
45	tatagagcaggaaaatccct		
46	agttgtcagcttaacactta		
47	ttacaacatggtcttctttg		
48	aggaaccatttatttagtca		
